# Construction of an adult barnacle (*Balanus amphitrite*) cDNA library and selection of reference genes for quantitative RT-PCR studies

**DOI:** 10.1186/1471-2199-10-62

**Published:** 2009-06-24

**Authors:** Tristano Bacchetti De Gregoris, Marco Borra, Elio Biffali, Thomas Bekel, J Grant Burgess, Richard R Kirby, Anthony S Clare

**Affiliations:** 1 School of Marine Science and Technology, Ridley Building, Newcastle University, Newcastle upon Tyne, NE1 7RU, England, UK; 2 Dove Marine Laboratory, Newcastle University, Cullercoats, Tyne and Wear, NE30 4PZ, England, UK; 3 Stazione Zoologica Anton Dohrn, Napoli, Villa Comunale, 80121, Italy; 4 Center for Biotechnology (CeBiTec), Bielefeld University, D-33594 Bielefeld, Germany; 5 School of Biological Sciences, University of Plymouth, Plymouth, PL4 8AA, England, UK

## Abstract

**Background:**

*Balanus amphitrite *is a barnacle commonly used in biofouling research. Although many aspects of its biology have been elucidated, the lack of genetic information is impeding a molecular understanding of its life cycle. As part of a wider multidisciplinary approach to reveal the biogenic cues influencing barnacle settlement and metamorphosis, we have sequenced and annotated the first cDNA library for *B. amphitrite*. We also present a systematic validation of potential reference genes for normalization of quantitative real-time PCR (qRT-PCR) data obtained from different developmental stages of this animal.

**Results:**

We generated a cDNA library containing expressed sequence tags (ESTs) from adult *B. amphitrite*. A total of 609 unique sequences (comprising 79 assembled clusters and 530 singlets) were derived from 905 reliable unidirectionally sequenced ESTs. Bioinformatics tools such as BLAST, HMMer and InterPro were employed to allow functional annotation of the ESTs. Based on these analyses, we selected 11 genes to study their ability to normalize qRT-PCR data. Total RNA extracted from 7 developmental stages was reverse transcribed and the expression stability of the selected genes was compared using *geNorm*, * BestKeeper *and *NormFinder*. These software programs produced highly comparable results, with the most stable gene being *mt-cyb*, while *tuba, tubb *and *cp1 *were clearly unsuitable for data normalization.

**Conclusion:**

The collection of *B. amphitrite *ESTs and their annotation has been made publically available representing an important resource for both basic and applied research on this species. We developed a qRT-PCR assay to determine the most reliable reference genes. Transcripts encoding cytochrome b and NADH dehydrogenase subunit 1 were expressed most stably, although other genes also performed well and could prove useful to normalize gene expression studies.

## Background

Many marine invertebrates have a pelagobenthic life cycle and biofouling by many of these species has a considerable economic impact in marine environments [1]. Consequently, it is essential to understand the mechanisms regulating the transition between the free-living planktonic larvae and the benthic adult stage. The barnacle *Balanus amphitrite *[2] is a sessile gregarious species that is a model organism for both fundamental and applied larval settlement studies due to its invasive behaviour, its worldwide distribution, and the relative simplicity of manipulating its reproduction in the laboratory. The life cycle of *B. amphitrite *is characterized by the presence of six planktonic naupliar stages (naupliar instar I-VI) followed by a non-feeding larval stage, the cyprid, that is specialized to explore the substratum in order to locate a suitable place for permanent attachment. A number of behavioural studies have shown that *B. amphitrite *cyprids respond to biotic and abiotic factors as they explore the substratum [3-5]. To date however, the paucity of genomic information available for this organism has hindered in-depth mechanistic studies of the surface colonization process.

Expressed sequence tag (EST) surveys are fundamental for discovering new genes [6] and they represent an essential step for the molecular characterization of the species of interest. In addition, EST-derived information supports genomic sequence annotation by suggesting intron/exon boundaries and the existence of previously undescribed transcription units; consequently, mRNA sequences are invaluable in comparative genomics [7]. We have therefore prepared an un-substracted cDNA library from adult *B. amphtrite *to identify the most expressed genes within the first few hundreds ESTs. We hope that the application of molecular probes developed from this EST library, in combination with standard methods for behavioural analysis, will allow us to better understand the timing and intensity of gene expression during different life history stages of *B. amphitrite*. Furthermore, few studies have investigated the regulation of the pelagobenthic life-cycle at a molecular level [8,9], despite its broad distribution in marine invertebrates [10]. Barnacles are good candidates to become a model system for this purpose, and the development of new molecular tool for these organisms could help to answer fundamental biological questions related to marine life.

Quantitative real-time PCR (qRT-PCR) is regarded as the most sensitive and reliable method to determine levels of mRNA transcription [11,12]. The application of qRT-PCR has proved particularly useful for comparative studies, where the expression of genes of interest (GOIs) in different samples is measured against the expression of endogenous reference genes (RGs). This normalization procedure is fundamental to minimize inherent variability introduced during the RNA extraction or the reverse transcription steps [13,14]. Ideally, RGs should both maintain a stable transcription level in all cells, tissues or individuals under investigation and should not be influenced by the experimental conditions. Unfortunately, many studies have shown that universal RGs for data normalization do not exist and for this reason, the selection of the best RGs should be validated for every new qRT-PCR assay [15].

Here, we describe the first characterization of the *B. amphitrite *transcriptome that is based on the creation of an EST library from adult individuals. The sequencing and annotation of 960 clones provides the background for further analysis of life-cycle regulation in this organism. We also established a qRT-PCR assay to monitor gene expression in different developmental stages and in individuals exposed to morphogenetic cues. The ability of 11 *B. amphitrite *transcripts to normalize qRT-PCR data was determined by comparing relative quantities obtained from cDNAs representing 14 different samples and 7 developmental stages. The software *geNorm *[16], *BestKeeper *[17]and *Normfinder *[18] were used to obtain an estimation of the expression stability of each gene and, by comparing the results, to identify the most suitable genes for qRT-PCR data normalization in *B. amphitrite*.

## Results and discussion

### Annotation of sequences from the ESTs library

**Figure 1:**
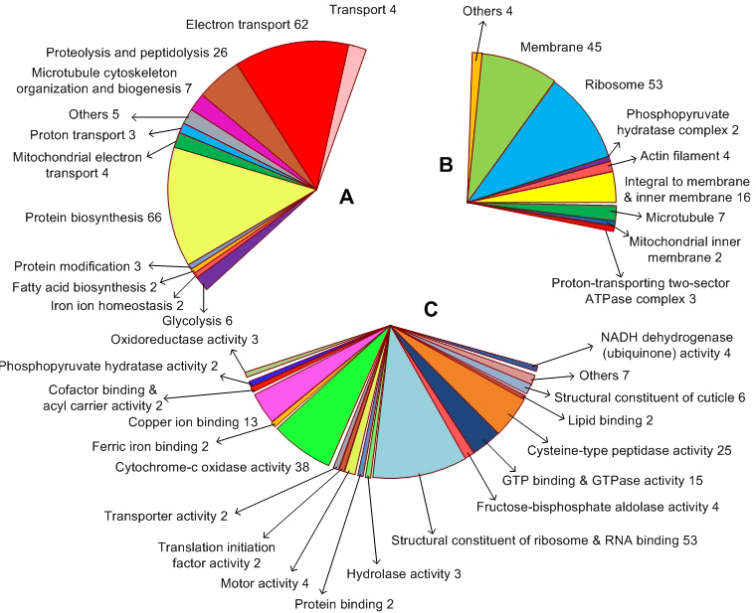
**Gene Ontology mapping for *Balanus amphitrite *proteins**. Relative distribution of ESTs within the three main subclasses existing in the GO classification: A) Biological process; B) Cellular component; C) Molecular function.

*Balanus amphitrite *is one of the most extensively studied barnacles and has been suggested as a candidate genetic model for larval settlement and metamorphosis [19]. Recently, Thiyagarajan and Qian used a proteomics approach to investigate settlement regulation in this organism [20]. However, they argued that the lack of deposited gene sequences hindered a full appreciation of their results. The creation of an adult *B. amphitrite *cDNA library, the sequence of 960 clones (of which 55 were excluded from our analysis as they showed insert length shorter than 50 bp) and the detection of 530 singlets and 79 tentative contiguous (TC) sequences (a summary of the EST survey is given in table 1) is thus an important first-step towards understanding the molecular ecology of this barnacle. Among the 609 different genes we report, 107 appeared to be of mitochondrial origin and 75 showed similarities with previously published ribosomal sequences. Gene ontology entries [21] revealed that the main categories of the genes found in our library appear to be involved in electron transport, protein biosynthesis, catalytic activity, metal ion binding, metabolism and the biogenesis of structural elements such as muscle and cuticle (Figure 1). Approximately 38% of the unique sequences we obtained have been functionally annotated and a corresponding gene name proposed, and informative annotation has been given for an additional 18%. Of the remaining ESTs, 8% had a BLAST match with uncharacterized transcripts and 37% showed no appreciable similarity to previously published sequences. This distribution of ESTs among known/uncharacterized/unknown genes does not differ substantially from that found in recent EST surveys on other marine invertebrates [22,23]. We also determined several transcripts that were highly similar to sequences derived from the deposited complete mitochondrial genomes of the two barnacles *Megabalanus volcano *and *Tetraclita japonica*.

**Table 1:** Classification of *Balanus amphitrite *ESTs

**Search method**	**Putative source**	**E-value**	**Annotation**	**N° of sequences**
Blast2n vs nt				457
InterPro				438
Blast2x vs SP				458
Blast2x vs KEGG				500
			Functionally annotated	449
			Unassigned ESTs	149
			Unknown ESTs	307
	Ribosomal RNA			123
	Mitochondrial DNA			202
	Genomic DNA			580
		< e-30		191
		> e-30		407

Vector clipped ESTs longer than 50 nucleotides and with an E-value > e-5 were functionally annotated whenever possible. Blast hits with E-values < e-5 were included in the unassigned proteins. Unknown ESTs in our dataset are those with no match in sequence databases. These ESTs were considered to be of genomic DNA origin as it is unlikely that the highly conserved mitochondrial gene would not result in a Blast hit.

Fragment assembly generated a total of 79 TC comprising 375 ESTs. Sequences belonging to 8 different TCs were particularly frequent in our library, with the most common being an unassigned mitochondrial gene partly similar to 16S rRNA (with 52 entries), followed by cytochrome c oxidase subunit I (31), cysteine proteinase (17), cytochrome c oxidase subunit II (14), cytochrome b (12), a ribosomal RNA internal transcribed spacer (12), cytochrome c oxidase subunit III (11) and the elongation factor 1-α (11). The longest TC generated was 1703 nucleotides and translated for the18S rRNA gene. Considering the 609 unique sequences we obtained, a total of 280 had a match in the NCBI nucleotide database. A taxonomic subdivision of the first hit produced by these 280 transcripts showed that 109 of them matched sequences from barnacles. The remaining sequences were represented among insects, vertebrates, arachnids, plants, fungi and various other groups (79, 53, 6, 8, 5 and 19 sequences, respectively). To annotate *B. amphitrite*'s genes, the proposed nomenclature for *Drosophila melanogaster *was used as a guide  and the corresponding gene symbol established in *D. melanogaster *was used when possible. However, in a slight departure, we decided to use the prefix mt- to identify mitochondrial genes.

### Validation of best reference genes for qRT-PCR

Our main interests focus on elucidating those genes involved in barnacle settlement. In this respect, qRT-PCR is particularly suitable to monitor how external cues, such as environmental variables, the presence of conspecific individuals or the occurrence of biofilm and/or of certain microorganisms, influence gene expression prior to and during settlement and metamorphosis. Since most of the annotated ESTs we found represent highly expressed housekeeping genes, this suggests that information from a few hundred clones derived from a cDNA library is sufficient to validate RGs for subsequent qRT-PCR studies.

We selected 14 potential RGs and designed PCR primer pairs to them (Table 2). These genes were chosen either because they were commonly used for other organisms or because they were often found in the EST library and their functional description indicated they might be useful candidate genes. Of the 14 primer pairs we tested, three (gapdh, ald, mlc1) produced PCR artefacts and therefore were discarded from our analysis without attempting to design new primer pairs for them as we considered a total of 11 genes to be adequate for our analysis. Primer efficiencies of the remaining 11 RGs varied between 84% and 100% (primer efficiency graphs are provided in additional file 1). To obtain reliable results it is important to achieve equivalent PCR efficiencies for the reference genes and the gene of interests. Therefore, pairs with a comparatively lower efficiency (84%) were kept in our analysis as they can be useful when investigating GOIs for which it is difficult to design primers with high efficiency.

**Table 2:** List of primers and reference genes under investigation

**Gene**	**Gene's symbol**	**Accession number**	**Forward primer**	**Reverse primer**	**Amplicon length**	**Primer efficiency**
Ubiquitin c	*ubc*	FM882773	GCGTCATAAGTTGCGGAGA	TCTTGGCCTTCACATTTTCA	106	100%

Fructose bisphosphate aldolase	*ald*	FM882346	TATGTCCCAGCGTTGTGCT	TGGCACCAGACCATTCATT	166	Non-specific products

NADH dehydrogenase subunit 1	*mt-nd1*	FM882393	CGGGCTGTTGCTCAAACTA	TTCGACAAAATCTTCCAATCT	102	100%

Tubulin alpha	*tuba*	FM882619	CCTGCTGGGAGCTGTATTGT	ACAACAGTGGGCTCCAAATC	169	94%

NADH dehydrogenase subunit 4L	*mt-nd4L*	FM882472	TTCTTGGTAGCTTCTGTGTGTG	TAGTCGGAACCATGTGATCG	80	100%

Tubulin beta	*tubb*	FM882322	ACCTCAGCCTGGTCATCATC	GGCTTTCCTCCACTGGTACA	165	84%

Cysteine protease 1	*cp1*	FM882273	GTTGAGCAGCACATGAAGGA	CGAACTCCTCAGAGGTCAGG	91	94%

Cytochrome b	*mt-cyb*	FM882286	GGACACTGCATGCTAATGGA	AGGCAGCAGCCATAGTCAAG	144	91%

Acyl carrier	*mt-acp*	FM882501	GATGTGGCGATTTGCTATCC	TTCTCCGGGTTGATCTTGTC	175	93%

Myosin 1-light chain	*mlc1*	FM882394	AAGGATGAGGTTGACGCCTA	ACCCTGGTCCTTGTCCTTCT	174	Presence of primer dimers

60s ribosomal protein L15	*rplL5*	FM882400	AAGCAGGGATACGCCATCTA	AGCTTCAGCTCGTTCACTCC	116	84%

Glyceraldehyde-3-phosphate dehydrogenase	*gapdh*	FM882736	TCTGCGGCTTACTTGTCCTT	ACTCGCACTCGAGCATCTTT	154	Non-specific products

Elongation Factor 1 alpha	*ef1a*	FM882302	GCCACAGGGATTTCATCAAG	TGGAGATACCAGCCTCGAAC	105	100%

Actin alpha	*act*	FM882301	CAGTCCAAGCGTGGTATCCT	CGCACGCAGCTCGTTGTAGA	114	100%

**Figure 2:**
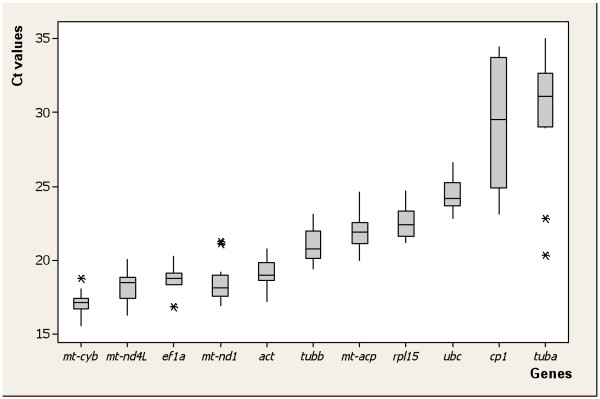
**Comparative expression of the analysed genes**. Box-and-whisker plot representing the expression level (threshold cycles) of candidate reference genes in *B. amphitrite *(*n *= 14). The box plot, obtained using the software Minitab, shows the smallest observation, lower quartile, median, upper quartile, largest observation and indicates Ct value that might be considered outliers.

**Figure 3:**
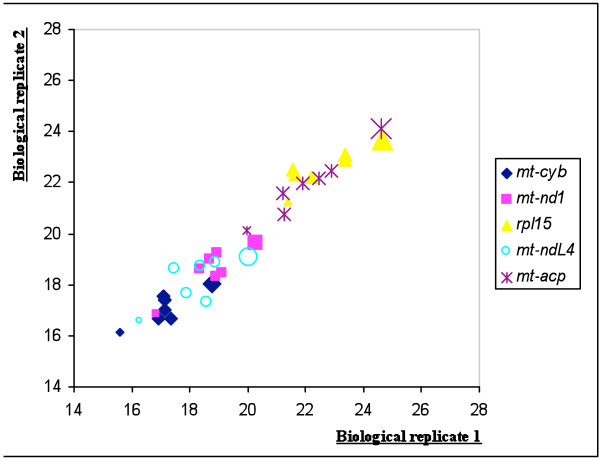
**Correlation between biological replicates for the five best reference genes**. The Ct values (adjusted to primer efficiencies) obtained for the seven developmental stages we analysed were plotted for the five best reference genes. The size of the shape indicates the developmental stage: the smallest shapes represent values from just-released nauplii, whereas the largest represent values obtained from adult barnacles.

The expression levels of RGs were obtained from qRT-PCR reactions in the form of threshold cycle (Ct) values (Figure 2). The 14 Ct values collected for each primer pair were derived from the two biological replicates of the seven developmental stages under investigation (raw Ct data are provided in additional file 2). These samples were initially considered independent and the data they generated were analyzed with *geNorm *[16], *BestKeeper *[17] and *NormFinder *[18] to determine the most steadily expressed genes. It can be argued that two biological replicates are not enough for statistical analysis, and this is the case for many biological systems (e.g. comparing different tissues, single individuals) where at least five replicates should be performed. In our investigation, however, the RNA was extracted from ten (the adult stage) to hundreds of pooled individuals, so that the RNA could be considered an average sample of the developmental stage analysed. In our opinion, the high correlation found between the two biological replicates, at least for the most stably expressed genes (figure 3), confirmed our expectations.

**Figure 4:**
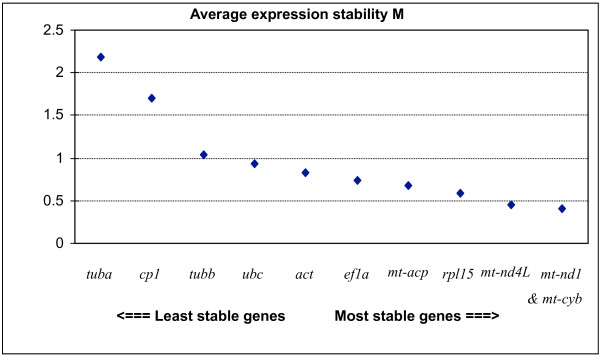
**Gene expression stability M of candidate reference genes calculated by *geNorm***. The *geNorm *program proceeds to the stepwise exclusion of the genes whose relative expression levels are more variable among samples. Data points represent the average expression stability values of remaining reference genes.

**Figure 5:**
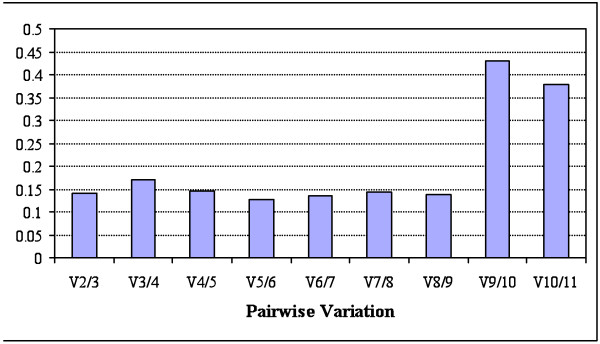
**Determination of the optimal number of reference genes for data normalization**. Bar values indicate the magnitude of the change in the normalization factor after the inclusion of an additional reference gene. The authors of *geNorm *suggest that V > 0.15 should be considered as the threshold to include an extra RG into the assay.

The software *geNorm *provides a ranking of the tested genes based on their stability measure (M), determining the two most stable RGs or a combination of multiple stable genes for normalization. The value M represents the mean pair-wise variation between a gene and all other tested candidates. The gene with the highest M is then excluded from the analysis and the calculation is repeated in a stepwise fashion that allows genes ranking until the best two genes are found. According to *geNorm*, the two most stable genes in our assay were *mt-nd1 *and *mt-cyb *(Figure 4), with an M value of 0.41. The threshold value M for considering a gene to be unsuitable for data normalization is suggested to be ≥ 1.5 [16]. Low values of the pair-wise variation V between two sequential normalization factors containing an increasing number of genes showed it was unnecessary to include another RG in our protocol (Figure 5). However, as *mt-nd1 *and *mt-cyb *are both contained in the mitochondrial genome, it may be advisable to include a nuclear gene in the normalization strategy. In this case, *geNorm *suggested that either *act *or *ef1a *should be used.

**Figure 6:**
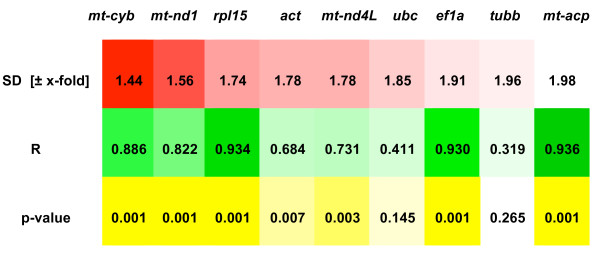
**Results from *BestKeeper *correlation analysis**. *BestKeeper *calculates the stability measure for each candidate gene and then ranks them from the most to the least stable (SD [± x-fold]). The coefficient of correlation (R) and the p-value measure the correlation between each gene and the *BestKeeper *index. For each variable presented in the figure (SD [± x-fold], R and p-value), genes that ranked comparatively better are highlighted with a more intense cell colour.

We excluded the highly variable *tuba *sequence from the analysis using *BestKeeper *[17], which can only consider 10 RGs. Since any gene calculated by *BestKeeper *to have a standard deviation >1 can be considered inconsistent, we also excluded *cp1 *(std dev of 3.69, descriptive statistics are provided in the additional file 3) from the final calculation of the *BestKeeper *index. This index is a representation of the average over/under-regulation of all genes together in every developmental stage. The RG that best correlated with the *BestKeeper *index was *mt-acp*, followed by *rpl15*, *ef1a *and *mt-cyb *(figure 6). In other words, *mt-acp *appeared to be the best candidate to represent the overall modulation of expression of the 9 RGs analysed. However, *mt-acp *was also the least stable gene (figure 6) and this was likely to influence the index to which it correlated. Nevertheless, the most stable gene, resulting from the *BestKeeper *analysis, was again *mt-cyb*, followed by *mt-nd1 *and *rpl15 *(figure 6).

**Figure 7:**
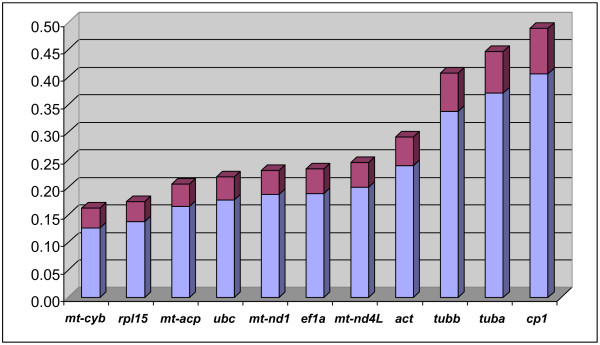
**Determination of the most stable reference genes using *NormFinder***. The *NormFinder *algorithm ranks the set of candidate normalization genes according to their expression stability in a given experimental design. Blue bars represent the stability values of our candidate genes, while purple bars indicate their standard error.

*NormFinder *attempts to identify the optimal normalization gene among a set of candidates and provides a measure of the stability of genes' expression in different groups and at the same time estimates any bias in the expression of the genes between the groups based on two-way ANOVA [18]. When all data were analysed together, the most stable RG candidates in our essay using *NormFinder *were *mt-cyb *(stability value = 0.127), *rpl15 *(0.137) and *mt-acp *(0.165), as shown in figure 7. We then repeated the analysis grouping samples by developmental stage to assess intergroup variability. In this case, the best genes that allows comparison of different developmental stages and/or treatments in *B. amphitrite*, which was the goal of this study, were *mt-cyb *(0.159), *mt-nd1 *(0.167) and *mt-acp *(0.168), suggesting that these are the most suitable genes for data normalization. A last examination was performed adding the data to *NormFinder *as two subgroups (the two biological replicates for each developmental stage). As a result, the software produced the same gene ranking by their stability values, showing a very low variability between replicates, which was also confirmed by further statistical analysis; Pearson correlation coefficients of biological replicates for all RGs tested ranged between 0.711 and 0.979, with 9 genes out of 11 showing a significant correlation at the 0.01 level (2-tailed). Although the use of only 7 data points may affect the examination, our results suggested that the implemented protocol is effective in capturing meaningful differences in gene expression throughout *B. amphitrite *development.

As a general consideration, although *geNorm*, *BestKeeper *and *NormFinder *have the same aim, they employ different strategies to calculate the most stable genes and it is unlikely that they will give the same results. For example, looking at the absolute ranking of best genes, *mt-acp *scored 5^th^, 9^th ^and 3^rd^. However, its stability values as determined by the tree software do not change substantially from that of the genes ranked closely (e.g. the value obtained by *BestKeeper *for *mt-acp *(9^th^) was 1.98 and that of *act *(4^th^) was 1.78). Finally, it was noted that Ct values for the best RGs tended to increase during the life cycle. This was particularly evident with the cDNA derived from adult barnacles, which required ~3 to 4 more cycles to reach the PCR exponential phase in comparison to the cDNA from larvae that had just hatched (Figure 3). While we cannot exclude the possibility that the genes analysed are down-regulated in the adult stage, this trend could also be explained by the presence of reverse transcription inhibitors that concentrate or are synthesized in later stages of *B. amphitrite *development, as RT-inhibitors are known to be one of the main sources of variability in qRT-PCR experiments [13]. Although the CT value shifts remained in an acceptable range, it may be advisable to include a reference assay to rule out the presence of inhibitors. This is commonly achieved by adding an aliquot of the RNA under investigation to a well characterised exogenous RNA and measuring the effect on the amplification of the cDNA derived from the latter [24,25].

## Conclusion

*Balanus amphitrite *is already established as a model organism to study the pelagobenthic life cycle. Here, we have presented the first cDNA library sequenced from adult *B. amphitrite*. We are currently generating three further normalized EST libraries for the developmental stages of nauplius I, cyprid and adult, and we estimate that another 15,000 sequences will be available soon. The addition of this genetic information will serve as an invaluable tool to investigate gene expression in barnacles. The three programs implemented to analyse qRT-PCR results indicated that *tuba*, *tubb *and *cp1 *are unsuitable genes for data normalization. They also showed that *mt-cyb *itself, and the pair *mt-cyb – mt-nd1*, were the genes expressed most stably throughout life cycle of *B. amphitrite*, and so we recommend their use as reliable reference genes in future qRT-PCR experiments. Other genes that performed well in our analyses were *mt-acp*, *rpl15*, *mt-nd4L*, *ef1a*, *ubc *and *act*.

## Methods

### *Balanus amphitrite*, culturing and RNA extraction

Wild *B. amphitrite *adults were collected from Beaufort, North Carolina, USA (courtesy of Prof. D. Rittschof). Brood stocks were maintained in semi-static culture in UV-irradiated, 10 μm filtered natural seawater. The adults were fed on newly-hatched *Artemia *sp. nauplii (Artemia International LCC, U.S.A.). To obtain barnacle nauplii, the adults were placed in a tank of fresh seawater and released larvae were attracted to a point light source and collected by pipette over a 2 h interval. Nauplii were cultured at the density of ~1 larva ml^-1 ^in an incubator at 28°C on a 12:12 light:dark cycle. The larvae were fed each day with 1 l of a *Skeletonema costatum *culture (~2 × 10^5 ^cells ml^-1^) until they reached the cyprid stage (approx. for 4–5 days). Cyprids were collected by filtering through a tier of filters (pore sizes of 350 and 250 μm) in order to discard undeveloped cyprids and microalgae, and stored at 6°C until use. The different developmental stages we studied were:

N-1) naupliar instar I – just hatched;

N-6) naupliar instar VI – three-eyed stage;

C-0) young cyprids – recently metamorphosed;

C-3) mature cyprids – these are standard larvae for settlement assays and they are maintained for 72 h at 6°C after the C-0 stage;

C-I) mature cyprids (same as C-3) that have been exposed to sea water containing 10^-5 ^M of 3-isobutyl-1-methylxanthine (IBMX) at 28°C for two hours [26];

J) juveniles collected ~24 hours after settlement onto glass slides;

A) adults.

All larvae were isolated under a dissecting microscope and placed in a 1.5 ml tube kept on ice. The tubes were centrifuged briefly and after the residual seawater was removed the larvae were resuspended in TRIzol (Invitrogen) prior to storage at -20°C. Settled juveniles were collected by scraping them off the glass slides using a sterile scalpel. For the adult stage, the pooled soft tissues of ten individuals were dissected and ground under liquid nitrogen prior to RNA extraction.

After the larval tissues were homogenized and crushed by pipetting and vigorous shaking, the total RNA was extracted from each biological replicate using 1 ml TRIzol. The extracted RNA was then stored in 1 ml of isopropanol at -20°C. Prior to cDNA synthesis the stored RNA was precipitated by centrifugation at 12,000 g for 5 min at 4°, washed twice with 1 ml of 70% ethanol and then resuspended in milliQ water. The RNA purity and quality were evaluated using a NanoDrop ND-1000 UV-Vis spectrophotometer (NanoDrop Technologies) and the quality was confirmed by gel electrophoresis (RNA picture provided in additional file 4).

### EST library creation and sequencing

Whole soft tissues of *B. amphitrite *were ground under liquid nitrogen and the total RNA was extracted using TRIzol as above. An EST library was then prepared by standard methods. Briefly, total RNA was first treated with DNase-1 to remove contaminating DNA, followed by a LiCl precipitation step. Messenger RNA was then purified from the total RNA pool prior to reverse transcription. The cDNA was prepared using the first strand synthesis primer 5'-GAGAGAGAGAGAGAGAGAGAACTAGT**CTCGAG**T^17^-3' (complementary to the poly-A mRNA tail), which contains an Xho-1 restriction site (in bold) to facilitate directional cloning of the 3' end of the ds-cDNA insert into the vector. The first strand synthesis used me5-dCTP rather than ordinary dCTP. Non-methylated dCTP was then used in the second strand reaction to make the complementary cDNA strand. This method prevents internal cleavage of the cDNA when the linker is digested subsequently with Xho-1. Prior to cloning, a double stranded linker containing a 5-Eco-R1 overhang (5'-OH-**AATT**CGGCACGAGG-3', overhang given in bold) was blunt-end ligated onto the ds-cDNA. The lack of phosphate on the 5' overhang for the Eco-RI linker prevented concatemerization during linker ligation (this was phosphorylated in a subsequent step). The linker-ligated cDNA was then digested with Xho-I and cloned directionally into the multiple cloning site of the plasmid vector pBluescript II SK+, previously linearised by digestion with the restriction enzymes Eco-RI and Xho-I. The library was cloned into DH5α cells and a total of 960 positive clones were randomly chosen to be sequenced. Plasmid DNA was prepared using a standard alkaline lysis plasmid prep [27]. Plasmids were sequenced using Sanger method (ABI BigDye Chemistry) and the sequencing reactions were run commercially on either an ABI-3700 capillary an ABI-3730 capillary or an ABI-377xl slab gel instruments using plasmid specific primers (Amplicon Express, USA). Both the quality-clipping and the subsequent base calling steps on the sequences were performed using the Phred13 software [28]. The average read lengths were 836 nucleotides for raw reads and 533 for high quality data.

### Clustering, assembly and functional annotation of the EST library

The bioinformatics analysis of the cDNA library BA23840 was performed using the sequence analysis and management system SAMS-2.0 [29]. We first applied a clustering step based on pair-wise comparison on the DNA level using the TIGR default parameters [30] to avoid redundancies in the dataset. Individual ESTs fall in the same cluster if they show a similarity of at least 95% over a region of not less than 40 bp in a pair-wise alignment and unmatched flanking regions must not exceed a length of 20 bp. Each cluster was then assembled using CAP3 [31], to produce 79 TCs and 530 singlets that were nearly free of redundancies and allowed the following functional analysis to be constructed within SAMS. After applying a modified GenDB [32] annotation pipeline consisting of a collection of standard bioinformatics tools including BLAST [33], HMMer [34] and InterPro [35] on each sequence, we applied Metanor [36], the GenDB automatic function prediction program. Regarding BLAST, while BlastX has been used for protein databases (NR, SP, Kegg and KOG), the BlastN algorithm was used for scanning the nucleotide database NT. By interpreting all the tool results we obtained, we created consistent functional annotations and assigned gene products, EC numbers, GO terms and KOG functional categories [37]. Finally, TCs and singlets were manually checked and gene names were given whenever possible. High quality ESTs were deposited in the EMBL database, accession numbers form FM882258 to FM883162. Assembly sets were also deposited to the EMBL under accession numbers FM994549 to FM994627. Access to sequences annotation via the SAMS interface will be provided upon request to the authors.

### Primer design

Tentative contiguous sequences (TCs) for RGs were analysed by Primer3 release 1.1.0 [38] using the following parameters: a) product size range: 80–180; b) primer size: min 16, opt 19, max 22; c) primer Tm: minimum 55°, optimum 60°, maximum 65°; d) primer GC%: minimum 40, optimum 50, maximum 60; e) all other parameters were left as the default. Oligonucleotides (synthesized by Invitrogen) were resuspended as stock solutions containing 0.7 pmol/μl of both the forward and reverse primers. The cDNA obtained from adult RNA was initially used to visualize the melting curve of PCR products and to determine the possible formation of PCR artefacts. PCR products were also sequenced to confirm specificity.

### cDNA synthesis, primer efficiencies and cycle parameter for qRT-PCR

We reverse transcribed 1 μg of total RNA from each sample for 20 min at 42°C with the QuantiTect kit (Qiagen). After the genomic wipe-out step and prior to the reverse transcription we collected 1 μl from each reaction to be later used as a negative-RT control to check for genomic contamination. Serial dilutions of 1:5, 1:10, 1:25, 1:50, 1:250, 1:500, 1:5000 and 1:50000 were then made from the initial 20 μl of adult cDNA. Each qRT-PCR experiments comprised 12.5 μl of Faststart SYBR green (Roche Diagnostics Ltd), 10.5 μl of stock primers (final concentration 0.3 μM each) and 2 μl of cDNA. Reactions were performed in sealed 96-well plates using a Chromo4 Research thermocyler and analyzed with the Opticon Monitor 3 software (BioRad).

The qRT-PCR thermal profile consisted of an initial step at 95°C for 5 min, followed by 40 cycles of 15 s, at 95°C and 1 min, at 60°C. A final elongation step at 72°C was included before the melting curve was determined by monitoring SYBR green fluorescence during the temperature ramp 60 to 95°C with an increase of 0.5°C and a hold of 1 s. We determined primer efficiencies using five cDNA dilution points for each primer pair that were chosen according to the expected expression level of the corresponding gene. Triplicates were tested for each dilution point and primer pair, together with a duplicate negative control that contained sterile water instead of cDNA. The resulting efficiency graphs are given in the additional file 1 accompanying this paper. To determine the best RGs, 2 μl of the cDNA diluted 1:50 were used for all 14 samples and primers tested.

### qRT-PCR data analysis

When required, raw Ct values were transformed to relative quantities by a comparative method based on the formula: 1/E^(Ct value-lowest Ct)^; where E is the primer efficiency and the lowest Ct refers to the smallest value obtained with each specific primer pair. The most stable RGs were then determined using software *geNorm *3.5 [16], *BestKeeper *[17] and *NormFinder *[18].

## Authors' contributions

TBDG is primarily responsible for the annotation of the ESTs, performed all qRT-PCR experiments and drafted the manuscript. MB participated in the experimental process and data analysis. EB provided expert input in designing the study. TB imported, clustered and assembled the ESTs into SAMS and helped with annotation. RRK provided the EST library. JGB and ASC supervised the study and critically revised the manuscript.

## Supplementary Material

Additional file 1 **Primer efficiency graphs**. The amplification efficiencies of the putative reference genes were calculated with the standard curve approach and derived from the formula E = 10^-1/slope^. Standard curves were generated using relative concentration vs. the threshold cycle (Ct). The linear correlation coefficient (R2) within 5 dilution points was calculated and the efficiencies, based on the slopes of the standard curves, ranged from 2.19 and 1.84. Click here for file

Additional file 2 **Raw Ct values obtained for all reference genes**. These values were obtained with the same threshold for every gene analysed and are not adjusted to the primer efficiency. Genes are organised in columns while developmental stages are displayed in rows. N1 = naupliar instar I; N6 = naupliar instar VI; C0 = cyprid day 0; C3 = cyprid day 3; CI = cyprid day 3 exposed to 10^-5 ^M of 3-isobutyl-1-methylxanthine; J = juveniles; A = adults. The numbers 1 and 2 following the developmental stage code identify for the two biological replicates. Click here for file

Additional file 3 ***BestKeeper *statistics**. The *BestKeeper *statistics for the 10 candidate genes initially analysed. The gene *cp1 *was then removed from further data processing with *BestKeeper*. Abbreviations: n = number of samples; geo Mean [CP] = geometric mean of threshold cycle (Ct); ar Mean [CP] = arithmetic mean of Ct; min and max [CP] = extreme values of Ct; std dev [± CP] = standard deviation of the Ct; CV [%CP] = coefficient of variance expressed as a percentage on the Ct level. Click here for file

Additional file 4 **Gel electrophoresis of *Balanus amphitrite *RNA**. Total RNA from *Balanus amphitrite *was run in a gel made of TBE and 1% agarose, and stained with ethidium bromide. The RNA (~1 μg) was run beside the RiboRuler™ high range RNA ladder (Fermentas), which contained 120 ng of RNA in each band. Band sizes (in number of bases) are given in the picture. Click here for file
