# Supplementary figures and images for: Construction of an adult barnacle (Balanus amphitrite) cDNA library and selection of reference genes for quantitative RT-PCR studies
Source: BMC Mol Biol. 2009 Jun 24;10:62. doi: 10.1186/1471-2199-10-62 (PMC2713238; doi:10.1186/1471-2199-10-62)

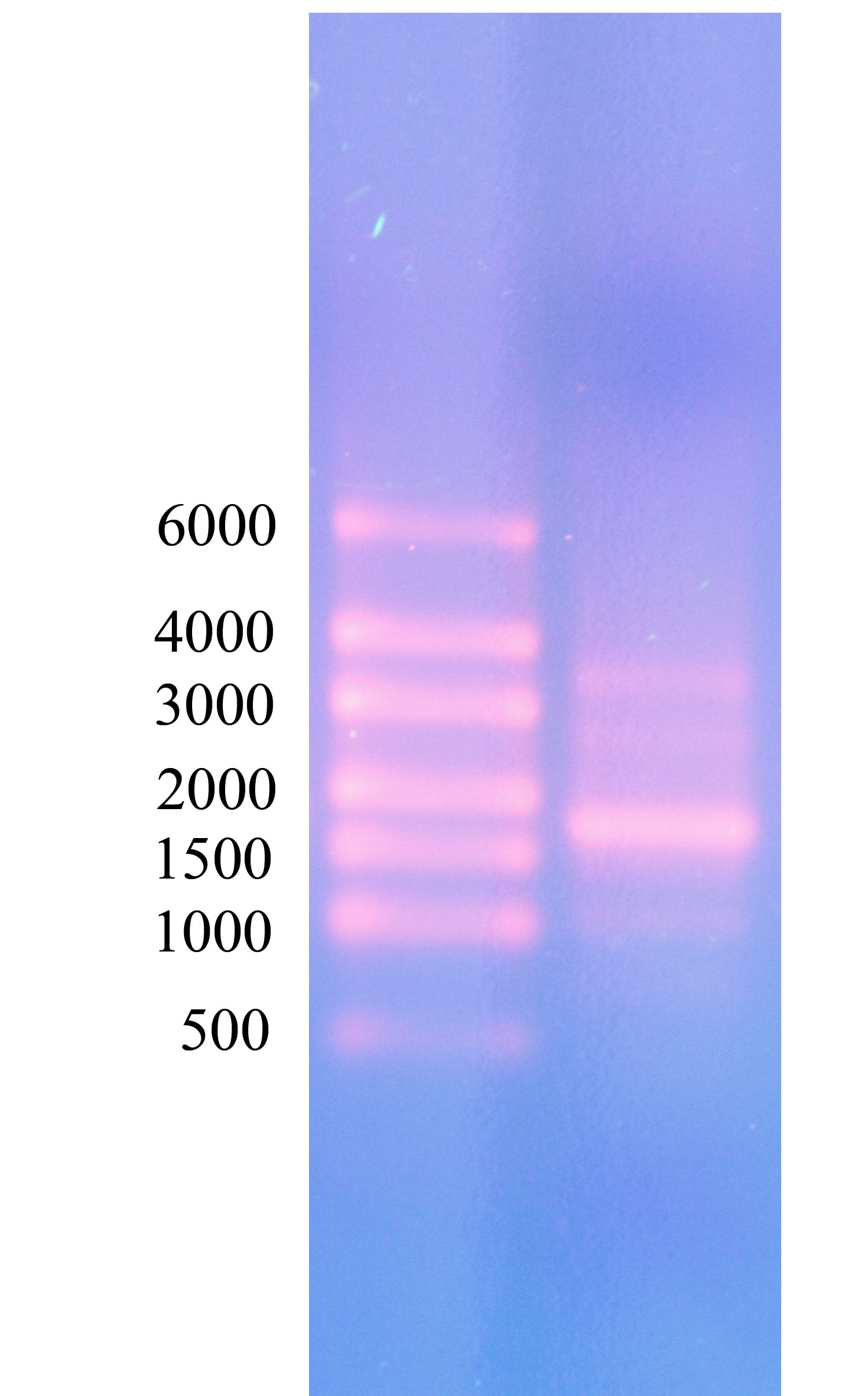

Supplement: Additional file 4 — Gel electrophoresis of Balanus amphitrite RNA. Total RNA from Balanus amphitrite was run in a gel made of TBE and 1% agarose, and stained with ethidium bromide. The RNA (~1 μg) was run beside the RiboRuler™ high range RNA ladder (Fermentas), which contained 120 ng of RNA in each band. Band sizes (in number of bases) are given in the picture. [file 1471-2199-10-62-S4.jpeg]
